# Nicotinic Acid Adenine Dinucleotide Phosphate (NAADP)-Mediated Calcium Signaling Is Active in Memory CD4^+^ T Cells

**DOI:** 10.3390/molecules29040907

**Published:** 2024-02-19

**Authors:** Anish Chakraborty, Ravindika Dissanayake, Katherine A. Wall

**Affiliations:** Department of Medicinal and Biological Chemistry, University of Toledo, Toledo, OH 43614, USA; fb4864@wayne.edu (A.C.); rdissan@rockets.utoledo.edu (R.D.)

**Keywords:** T cell receptor, NAADP, nicotinic acid adenine dinucleotide phosphate, calcium signaling, Ned-19, memory T cells

## Abstract

Nicotinic acid adenine dinucleotide phosphate (NAADP), identified as one of the most potent calcium-mobilizing second messengers, has been studied in different eukaryotic cell types, including lymphocytes. Although aspects of NAADP-mediated calcium release in lymphocytes are still under debate, the organelles pertaining to NAADP-mediated calcium release are often characterized as acidic and related to lysosomes. Although NAADP-mediated calcium release in different subsets of T cells, including naïve, effector and natural regulatory T cells, has been studied, it has not been widely studied in memory CD4^+^ T cells, which show a different calcium flux profile. Using a pharmacological approach, the effect of Ned-19, an NAADP pathway antagonist, on the involvement of NAADP in TCR activation in murine memory CD4^+^ T cells and their downstream effector functions, such as proliferation and cytokine production, was studied. According to this study, Ned-19 inhibited TCR-mediated calcium flux and its downstream effector functions in primary memory CD4^+^ T cells. The study also revealed that both extracellular and intracellular calcium stores, including endoplasmic reticulum and lysosome-like acidic calcium stores, contribute to the TCR-mediated calcium flux in memory CD4^+^ T cells. NAADP-AM, a cell permeable analogue of NAADP, was shown to release calcium in memory CD4^+^ T cells and calcium flux was inhibited by Ned-19.

## 1. Introduction

In lymphocytes, Ca^2+^ signals regulate various cellular functions, including differentiation, gene transcription and effector functions, such as vesicle exocytosis of lytic granules consisting of perforin and granzymes in cytotoxic T cells (CTLs) [1,2]. In T lymphocytes, T cell receptor (TCR) stimulation leads to activation of PLC-γ (phospholipase C gamma), which breaks down phosphatidylinositol-3,4-biphosphate (PIP_2_) into two hydrolytic products, inositol-1,4,5-triphosphate (IP_3_) and diacylglycerol (DAG). The endoplasmic reticulum (ER) Ca^2+^ store depletion caused by the binding of IP_3_ to IP_3_ receptors on the membranes of ER triggers the opening of store-operated Ca^2+^ release-activated Ca^2+^ (CRAC) channels via stromal interaction molecule (STIM) 1/2-ORA1 interaction and elevates the intracellular Ca^2+^ concentration [2,3]. Along with IP_3_, TCR stimulation leads to generation of other second messengers, cyclic adenosine diphosphate ribose (cADPR) and nicotinic acid adenine dinucleotide phosphate (NAADP), which contribute to the rise in the intracellular Ca^2+^ concentration [3].

NAADP microinjection into intact Jurkat T cells was reported to stimulate Ca^2+^ signaling in a dose-dependent manner. The role of NAADP in Ca^2+^ release in Jurkat T lymphocytes was further elaborated by its self-inactivation property, where microinjection of a high concentration of NAADP (10 µM) along with cADPR/IP_3_ completely inhibited the cADPR/IP_3_-mediated Ca^2+^ flux. NAADP (10 µM) microinjection prior to stimulation also completely blocked the anti-human CD3 monoclonal antibody OKT3-induced TCR/CD3-mediated Ca^2+^ signaling [4]. Gasser et al. measured the cellular NAADP following stimulation of Jurkat T cells with OKT3 using an enzyme assay and showed that the cellular NAADP rise followed biphasic kinetics and the NAADP rise was inhibited by tyrosine kinase inhibition [5]. NAADP-mediated Ca^2+^ signaling has been shown to be regulated by different ion channels/receptors, such as the ryanodine receptor (RyR), two-pore channels (TPC), transient receptor potential (TRP) channels, etc., and that varies from cell to cell preparation [6]. Using photoaffinity labeling, the existence of different NAADP-binding proteins in different cell preparations, including sea urchin egg, T-lymphocytes and mammalian cells (SKBR3, HEK293, mouse pancreas), has been established [7,8,9]. Two NAADP-binding proteins that appear to control Ca^2+^ release via two-pore channels or RyR have been identified [10,11,12,13]. There are controversies regarding NAADP-mediated release of intracellular Ca^2+^ either from endo/lysosomes/acidic stores or ER stores or both [6,14]. NAADP causes Ca^2+^ release from reserve granules (lysosome-associated organelles) in sea urchin eggs [15] and from endoplasmic reticulum via ryanodine receptors in Jurkat T lymphocytes [16,17,18]. The reduction of NAADP-induced Ca^2+^ spikes in the presence of either 8-NH_2_-cADPR (cADPR antagonist) or heparin (IP_3_ antagonist) in pancreatic acinar cells delineated the concept of “Ca^2+^-induced Ca^2+^ release”, where NAADP-mediated locally released Ca^2+^ triggered global Ca^2+^ signal by acting via the IP_3_ and ryanodine receptors [19]. Specific proteins, such as the progesterone receptor membrane component 1 (PGRMC1), can facilitate endosome/ER interaction [20].

Ned-19, which was discovered via a virtual screening strategy, antagonized NAADP-mediated Ca^2+^ flux in intact sea urchin eggs and NAADP-mediated Ca^2+^ spiking in mouse pancreatic beta cells [21]. It is thought to be an antagonist of the NAADP signaling pathway, although its direct binding partners have not been demonstrated. Using Ned-19 antagonism, Ali et al. have shown that lysosome-like acidic stores and ER stores serve as the principal and contributory sources, respectively, of NAADP-mediated Ca^2+^ release in naïve and effector mouse T cells [22]. In this study, since the profiles of Ca^2+^ flux in memory cells are quite different from those in naïve and effector T cells, we asked whether NAADP-mediated Ca^2+^ flux exists in memory CD4^+^ T cells. If it exists, what are the sources of NAADP-mediated Ca^2+^ release. Furthermore, we explored what downstream effector functions of memory CD4^+^ T cells would be controlled by NAADP-mediated Ca^2+^ signaling.

## 2. Results

### 2.1. Ned-19 Inhibits Memory CD4^+^ T Cell Proliferation Whereas BZ194 Does Not Suppress the Proliferation 

**Figure 1:**
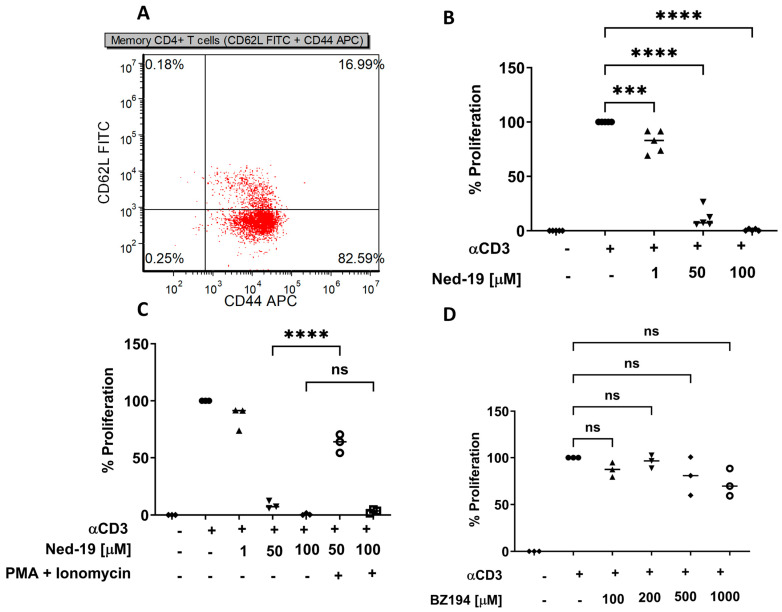
Ned-19 but not BZ194 inhibits memory CD4^+^ T cell proliferation. (**A**) Dot plot of isolated memory CD4^+^ T cells (effector-CD62L^low^CD44^high^ and central-CD62L^high^CD44^high^ memory CD4^+^ T cells. (**B**) Ned-19 inhibits memory CD4^+^ T cell proliferation. Memory CD4^+^ T cells (10^5^/well) were stimulated using 10 µg/mL anti-CD3 or phosphate buffered saline (PBS) in the presence of Ned-19 (various concentrations) or dimethyl sulfoxide (DMSO) added 1 h prior to stimulation. Proliferation was evaluated via [^3^H]-thymidine incorporation. In all cases, the average % proliferation was calculated, setting memory CD4^+^ T cells stimulated by anti-CD3 in DMSO after background correction as 100% proliferation. The data were the mean value of 5 individual experiments. (**C**) Proliferative capacity of treated memory CD4^+^ T cells. Cells were incubated with Ned-19 or DMSO for 1 h, followed by addition of PMA (100 ng/mL) and ionomycin (500 ng/mL) to 50 and 100 µM Ned-19 treated samples, followed by 10 µg/mL anti-CD3 or PBS. The data were the mean value of 3 individual experiments. (**D**) BZ194 does not inhibit memory CD4^+^ T cell proliferation. Cells were incubated with BZ194 or DMSO for 5 h, followed by 10 µg/mL anti-CD3 or PBS. The data were the mean value of 3 individual experiments. (**B**) Kolmogorov–Smirnov test, (**C**,**D**) Shapiro–Wilk Test, ANOVA, **** *p* < 0.0001, *** *p* < 0.001, ns = not significant.

Polyclonal memory CD4^+^ T cells were isolated from mouse spleen using a memory CD4^+^ T cell isolation kit (Stemcell Technologies, Vancouver, BC, Canada). As shown in Figure 1A, isolated memory CD4^+^ T cells consist of 16.99% central memory CD4^+^ T cells (CD62L^high^CD44^high^) and 82.59% effector memory CD4^+^ T cells (CD62L^low^CD44^high^). As shown in Figure 1B, Ned-19 inhibited anti-mouse CD3-driven proliferation in a concentration-dependent manner. The significant inhibitory effect of Ned-19 toward proliferation at 50 and 100 µM concentrations raised a question regarding the possible cytotoxic effects of Ned-19 at those two concentrations. In order to test the responsiveness of the Ned-19 treated cells, after the 1 h incubation of cells with 50 and 100 µM of Ned-19, 100 ng/mL phorbol-12-myristate-13-acetate (PMA) and 500 ng/mL ionomycin were added before anti-CD3 stimulation. As shown in Figure 1C, addition of PMA and ionomycin to the cell suspension incubated for 1 h with 50 µM Ned-19 showed restoration of proliferation. Addition of PMA and ionomycin to the 100 µM Ned-19-treated cells did not reverse the inhibition. Therefore, concentrations of Ned-19 at 100 µM and above are considered to irreversibly inhibit proliferation.

Dammerman et al. have synthesized 3-carboxy-1-octylcarbamoylmethyl-pyridinium bromide (BZ194) and reported BZ194 (NAADP antagonist acting via RyR1 inhibition)-mediated inhibition of the downstream effector functions of Ca^2+^ signaling in antigen-specific CD4^+^ effector T cells [23]. We studied the effect of BZ194 on memory CD4^+^ T cell proliferation. After isolation, memory CD4^+^ T cells were incubated with various concentrations of BZ194 or DMSO for 5 h and then the cells were stimulated with anti-CD3 (10 µg/mL) and proliferation was evaluated. As shown in Figure 1D, BZ194 does not have any effect on memory CD4^+^ T cell proliferation. It may be that a longer incubation is necessary to see an effect, as Dammerman et al. cultured their rat T cell clones overnight in BZ194. Our memory T cells did not survive overnight culture when unstimulated, so the conditions could not be exactly duplicated.

### 2.2. Ned-19 Inhibits Cytokine Production by Memory CD4^+^ T Cells

**Figure 2:**
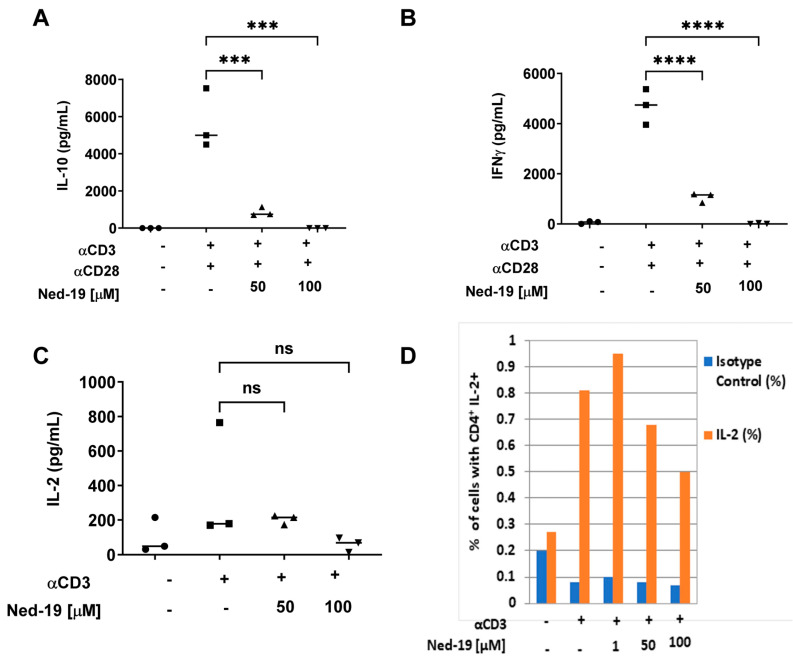
Ned-19 inhibits cytokine production by memory CD4^+^ T cells. (**A**) Memory CD4^+^ T cells (10^5^/well) were incubated for 1 h with Ned-19 and then stimulated with anti- CD3 (2 µg/mL) and soluble anti-CD28 (1 µg/mL) for 96 h. Supernatants were assayed for IL-10 via ELISA. Data representative of 3 experiments. (**B**) IFN-γ ELISA of supernatants from (**A**). Shapiro–Wilk test, ANOVA, **** *p* < 0.0001, *** *p* < 0.001. (**C**) Treated cells were stimulated with anti- CD3 (10 µg/mL) for 48 h. The supernatants were assayed for IL-2 via ELISA. Kruskal–Wallis test, ns = not significant. (**D**) Ned-19 inhibits accumulation of IL-2 in memory CD4^+^ T cells. Memory CD4^+^ T cells were incubated for 1 h with Ned-19 or DMSO and then stimulated by anti-CD3 for 6.5 h, followed by addition of 1000× Brefeldin A solution (1:1000) and incubated for another 7.5 h. Thereafter, intracellular IL-2 staining was done after fixation and permeabilization. The figure shows the % of cells with CD4^+^IL-2^+^ or CD4^+^Isotype^+^ as obtained by means of flow cytometry. Gating and cytometry data are provided in Figure S1. The experiment was repeated twice with similar results.

Ali et al. have shown Ned-19-mediated inhibition of interleukin (IL)-2 secretion by naïve CD4^+^ and CD8^+^ T cells and of interferon (IFN)-γ secretion by naïve CD8^+^ T cells stimulated using anti-CD3 and anti-CD28 [22]. We investigated the effect of Ned-19 on IL-2, IL-10, and IFN-γ secretion by stimulated memory CD4^+^ T cells. As shown in Figure 2, Ned-19 exhibited significant inhibition of IL-10 (A) and IFN-γ (B) secretion by memory CD4^+^ T cells. Ned-19 partially inhibited IL-2 (C) secretion by memory CD4^+^ T cells, although this was not statistically significant and the overall production was low. Partial inhibition of IL-2 secretion by Ned-19 prompted us to ask whether this was due to intracellular accumulation of IL-2. IL-2 secretion was inhibited using brefeldin A, an intracellular protein transport inhibitor. The addition of brefeldin A during the last hours of the in vitro cell activation phase inhibited protein transport to the Golgi complex (GC) and thereby proteins accumulated in the ER. The deposited proteins could be detected using flow cytometry. Gating and cytometry data are provided in Figure S1. As shown in Figure 2D, intracellular IL-2 accumulation decreased by about 40% with 100 µM Ned-19 in anti- CD3-stimulated memory CD4^+^ T cells. Therefore, reduction of IL-2 secretion was due to a reduction of synthesis and not a lack of secretion of the cytokine.

### 2.3. Ned-19-Mediated Inhibition of Ca^2+^ Flux in Memory CD4^+^ T Cells and Naïve CD4^+^ T Cells

**Figure 3:**
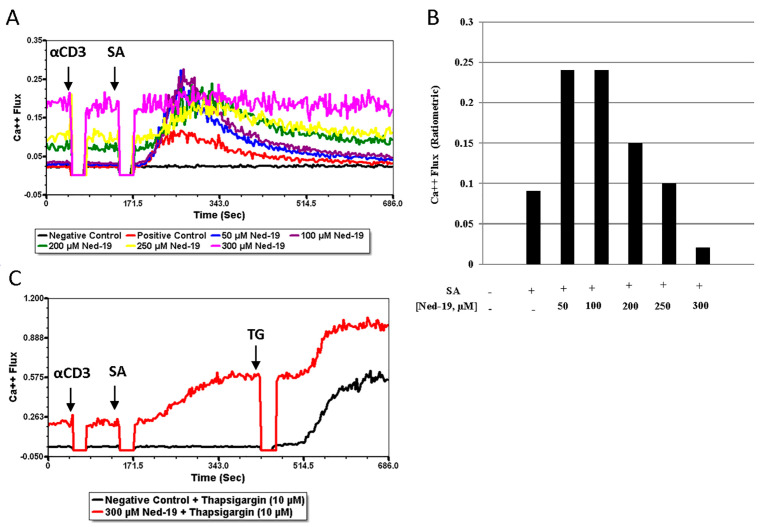
Ned-19 effect on Ca^2+^ flux in isolated memory CD4^+^ T cells. Fluo-4 AM and Fura Red-labeled memory CD4^+^ T cells (10^5^/tube) were incubated at 37 °C and 5% CO_2_ with DMSO or increasing concentrations of Ned-19 for 1 h. Ca^2+^ flux was measured in a flow cytometer via addition of anti-mouse CD3 at 50 s followed by PBS (negative control) or Streptavidin (SA) addition at 2 min 30 s. Acquisition was briefly interrupted for reagent addition. (**A**) Overlaid time courses of ratiometric Ca^2+^ flux of different memory CD4^+^ T cell samples with DMSO or increasing concentrations of Ned-19. The data are representative of 2 separate experiments. (**B**) Peak amplitude of ratiometric Ca^2+^ flux of (**A**). (**C**) Checking responsiveness of negative control and 300 μM Ned-19 with addition of 10 μM thapsigargin (TG) at 7 min 15 s.

As Ned-19 inhibited several downstream effector functions of the Ca^2+^ signaling of memory CD4^+^ T cells, we performed Ca^2+^ flux experiments with isolated memory CD4^+^ T cells. The NAADP antagonist Ned-19 inhibition of receptor-mediated Ca^2+^ flux in naïve and effector CD4^+^ T cells [22] incited us to check whether Ned-19 antagonized receptor-mediated Ca^2+^ flux in memory CD4^+^ T cells. Fluo-4 AM and Fura Red-labeled memory CD4^+^ T cells were incubated for 1 h with DMSO or various concentrations of Ned-19, and the Ca^2+^ flux, defined as the FL1/FL3 ratio, was analyzed using a flow cytometer. The cells were stimulated by adding biotinylated anti-CD3 at 50 s and streptavidin or PBS at 2 min 30 s. As shown in Figure 3A,B, 50 to 200 µM Ned-19 actually increased the Ca release upon stimulation. Treatment with 300 µM Ned-19 inhibited receptor-mediated Ca^2+^ flux in memory CD4^+^ T cells. 

Pitt et al. have previously demonstrated that with direct application to TPC2, Ned-19 stimulates NAADP-mediated TPC2 activation at low nanomolar concentrations, such as 30 to 100 nM, and inhibits (non-competitively) at 1 µM [24]. Given that the internal concentration of Ned-19 is not known, it is possible that Ned-19 showed a stimulatory effect on Ca^2+^ flux at 50–200 µM concentrations and an inhibitory effect at 250–300 µM. The increased baseline at 200–300 µM concentrations of Ned-19 prior to receptor-mediated stimulation could be due to a Ned-19-mediated increase in the intracellular Ca^2+^ concentration. Since we did see a higher baseline with more Ned-19, we checked the responsiveness of the cells via the addition of sarco/endoplasmic reticulum Ca^2+^-ATPase inhibitor thapsigargin (10 µM) at around 7 min 15 s to both the negative control and the 300 µM Ned-19 treated cells (Figure 3C). Both populations responded similarly. The stimulation of Ca^2+^ flux at low Ned-19 concentrations and the increase in the baseline at higher concentrations distinguish memory cells from naïve cells, as previously studied [22]. In the experiments reported in [22], spleen CD4^+^ T cells, which consisted of 86% naïve cells, were analyzed using digital fluorescence imaging microscopy.

### 2.4. Ca^2+^ from External Medium and Intracellular Stores Both Contribute to Receptor-Mediated Ca^2+^ Flux in Memory CD4^+^ T Cells

**Figure 4:**
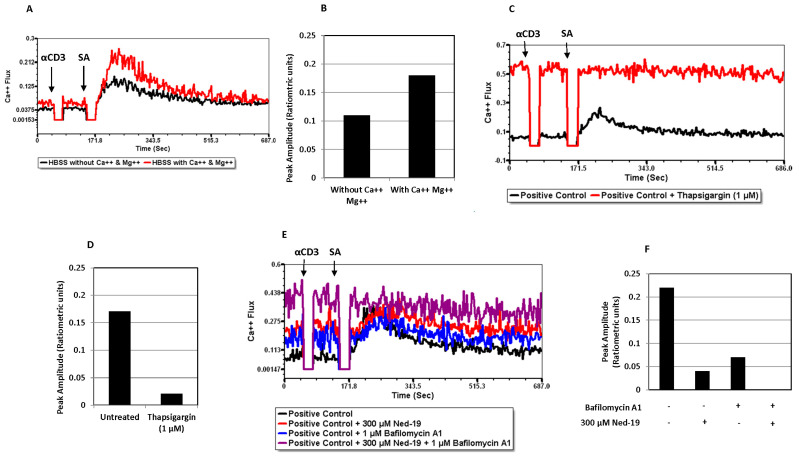
Contribution of external medium and internal stores (ER and lysosomes or acidic stores) in receptor-mediated Ca^2+^ flux of memory CD4^+^ T cells. In all the figures, memory CD4^+^ T cells (10^5^/tube) were stimulated via biotinylated anti-mouse CD3 and streptavidin. (**A**) Overlaid time courses of ratiometric Ca^2+^ flux in HBSS with or without Ca^2+^ and Mg^2+^ as FACS medium. (**B**) Peak amplitudes from (**A**). (**C**) Overlaid time courses of ratiometric Ca^2+^flux after 30 min of incubation with/without 1 μM thapsigargin. (**D**) Peak amplitudes from (**C**). (**E**) Overlaid time courses of ratiometric Ca^2+^flux after 30 min and 1 h incubation with/without bafilomycin A1 and with/without 300 μM Ned-19, respectively. (**F**) Peak amplitudes from (**E**). αCD3 = biotinylated anti-mouse CD3 and SA = streptavidin.

We determined the relative contribution of the external medium and intracellular Ca^2+^ stores in the observed Ca^2+^ flux. As shown in Figure 4A,B, the enhancement of the receptor-mediated Ca^2+^ flux in Hank’s balanced salt solution (HBSS) with Ca^2+^ and Mg^2+^ as compared to Ca^2+^ flux in HBSS without Ca^2+^ and Mg^2+^ revealed the contribution of external Ca^2+^ in the receptor-mediated Ca^2+^ flux in memory CD4^+^ T cells. Thapsigargin, a sarco/endoplasmic reticulum Ca^2+^ ATPase (SERCA) inhibitor, depletes ER Ca^2+^ stores and enhances the intracellular Ca^2+^ concentration. Thapsigargin pre-treatment provided almost complete inhibition of Ca^2+^ flux, indicating that ER stores contribute to the receptor-mediated Ca^2+^ flux in memory CD4^+^ T cells (Figure 4C,D). The higher baseline in Figure 4C in the thapsigargin-treated sample as compared to the control one presumably reflects the higher Ca^2+^ concentration in thapsigargin-treated cells. Pre-treatment with bafilomycin A1, an inhibitor of the vacuolar proton pump (H^+^-ATPase), resulted in partial inhibition, indicating that lysosomes or acidic stores also contribute to the receptor-mediated Ca^2+^ flux in memory CD4^+^ T cells (Figure 4E,F). Since TPC largely function to release Ca^2+^ from acidic stores [25], these data support a role for TPC in T cell Ca^2+^ release.

### 2.5. Imaging of Lysosomes of Memory CD4^+^ T Cells

**Figure 5:**
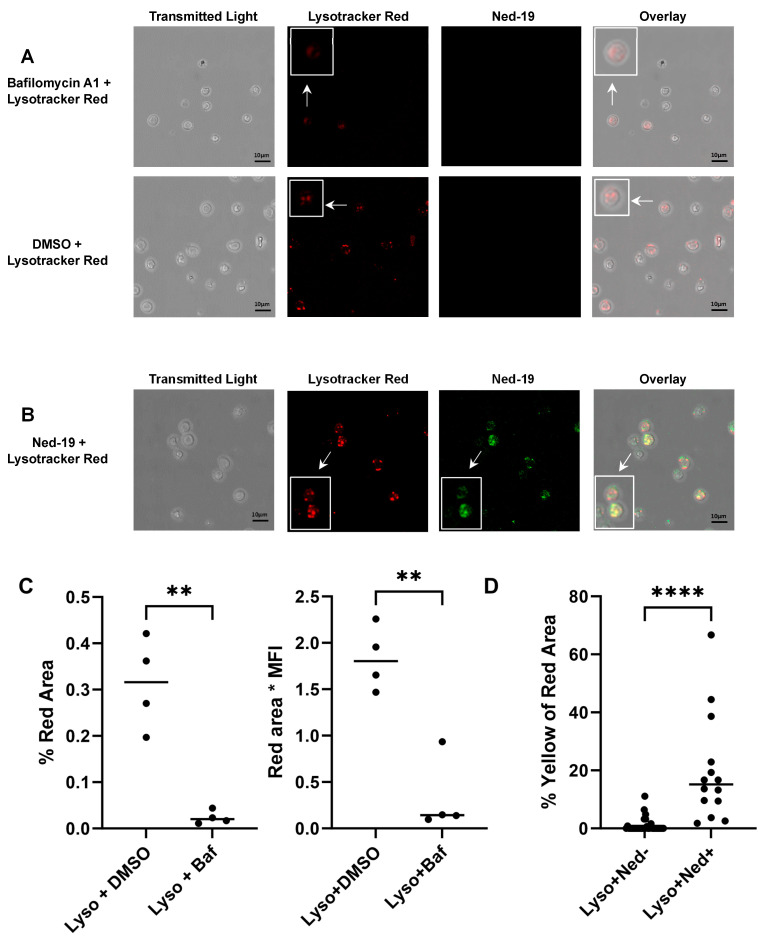
Imaging of lysosomes of memory CD4^+^ T cells (5 × 10^5^/dish). (**A**) Confocal image of lysosomes after 30 min of incubation with bafilomycin Al (Baf, 1 µM) or DMSO with Lysotracker Red DND-99 (Lyso, 60 nM). (**B**) Confocal image of lysosomes after 1 h of incubation with Ned-19 (100 µM) and Lysotracker Red DND-99 (60 nM). In (**A**,**B**), the arrowhead portions of the images have been boxed as enlarged for convenience. (**C**) Quantitation of lysotracker red fluorescence as the % of the total image (left) and red area times mean fluorescence intensity (MFI, right) for each image, 4 images, 2 experiments, Lyso + DMSO *n* = 55 cells, Lyso + Baf n = 39 cells. Shapiro–Wilk test, unpaired *t* test, ** *p* < 0.01. (**D**) Quantitation of the overlap of Ned-19 (Ned) green with Lysotracker Red as the % yellow of the red area for each cell individually, 4 images, 2 experiments, Lyso + Ned19- n = 26 cells, Lyso + Ned+ n = 14 cells. Mann–Whitney test, **** *p* < 0.0001. Bar = 10 µm.

The inhibition of memory CD4^+^ T cell Ca^2+^ flux by bafilomycin A1, as shown in Figure 4E,F, suggests a role for lysosomes in the release of Ca^2+^ by memory T cells. Multiple investigators, including Ali et al., have shown that bafilomycin A1 affects the pH gradient of lysosomes and therefore lysosomal function in many cell types [22,26,27]. The effect of bafilomycin on memory T cells was confirmed by capturing confocal images of intact lysosomes after staining with Lysotracker Red DND-99 in the presence or absence of bafilomycin A1. As shown in Figure 5A, the image showed the disruption of the pH gradient of the lysosomes by bafilomycin A1 and loss of the Lysotracker Red signal. The loss of Lysotracker staining was quantitated in Figure 5C as the % red area/total image area and read area times the mean fluorescence intensity for control and bafilomycin A1-treated cells. The exact mechanism of Ned-19 action is not known, but it is thought to interact directly or indirectly with TPC on acidic organelles [21,28]. We previously showed Ned-19 access to lysosomes in other T cells [22]. Therefore, we asked if we could localize Ned-19 within treated memory T cells. As shown in Figure 5B, the confocal image of memory CD4^+^ T cells incubated for 1 h with Ned-19 and Lysotracker Red DND-99 shows partial co-localization of Ned-19 and Lysotracker Red DND-99. The area of each color was determined in individual cells using ImageJ. Figure 5D shows that, on average, 15.15% of the lysosomal red area contained Ned-19 (yellow overlap), whereas 0.0% of the red area of the control cells was yellow. Therefore, Ned-19 was able to enter the lysosomes and could interact with lysosomal proteins such as TPC. The image also shows that Ned-19 did not disrupt the pH gradient of the lysosomes.

### 2.6. Ned-19 Inhibits NAADP-AM-Mediated Ca^2+^ Flux in Memory CD4^+^ T Cells

**Figure 6:**
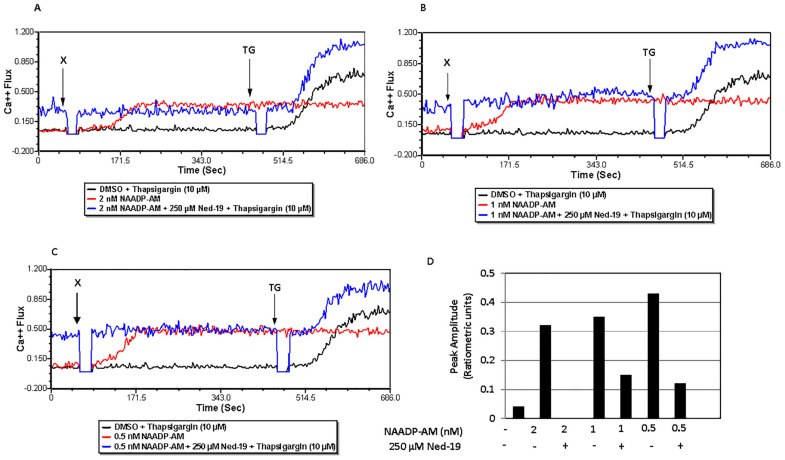
Ned-19 inhibits NAADP-AM-mediated Ca^2+^ flux in memory CD4^+^ T cells. (**A**–**C**) are overlaid time courses of ratiometric Ca^2+^ fluxes of memory CD4^+^ T cells (10^5^/tube), stimulated by DMSO or NAADP-AM [2, 1 and 0.5 nM NAADP-AM in (**A**–**C**), respectively] after incubating cells 1 h with or without Ned-19. The cell viability of selected samples was checked via they addition of thapsigargin (10 µM) at 7 min 30 s. X = DMSO or NAADP-AM and TG = thapsigargin, (**D**) peak amplitude after 1 h of incubation with or without 250 µM Ned-19.

After observing the inhibitory effects of Ned-19 in memory CD4^+^ T cells on downstream effector functions, we checked if Ned-19 could suppress direct NAADP-mediated Ca^2+^ flux. First, various concentrations of NAADP in PBS were added directly to memory CD4^+^ T cells during the Ca^2+^ flux measurement, but that did not cause any Ca^2+^ flux, possibility due to impermeability into the cell. Thereafter, liposomal delivery of NAADP was tried; however, it also did not cause any Ca^2+^ flux. Parkesh et al. have reported a cell-permeant analogue of NAADP, NAADP-AM, which readily goes inside the cell and causes Ca^2+^ flux [29]. Therefore, Ca^2+^ flux via addition of NAADP-AM was attempted. Although the effects were variable, 2 nM, 1 nM and 0.5 nM NAADP-AM addition at 1 min did generate Ca^2+^ flux, which was inhibited in each case by previous 1 h incubation with 250 µM Ned-19 (as shown in Figure 6). The responsiveness of 250 µM Ned-19-inhibited memory CD4^+^ T cells was checked via the addition of thapsigargin (10 µM) at 7 min 30 s.

### 2.7. Ned-19 Inhibits Nuclear Translocation of NFAT and NF-κB

**Figure 7:**
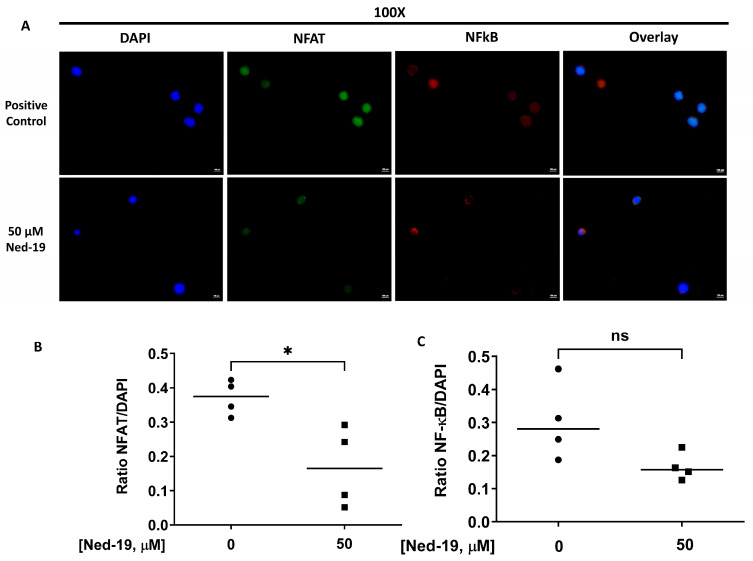
Ned-19 inhibits nuclear translocation of NFAT and NF-κB. Memory CD4^+^ T cells (3 × 10^5^/well) were stimulated by anti-mouse CD3 for 48 h after 1 h of incubation in the absence or presence of 50 µM Ned-19. After 48 h, immunohistochemistry analysis was performed. (**A**) DAPI (blue), NFAT (green), NF-κB (red) and overlaid images of staining. (**B**,**C**) Quantification of NFAT and NF-κB in the nucleus was done by taking the ratio of integrated intensity of NFAT (**B**) or NF-κB (**C**) to DAPI. n = 4/group, Shapiro–Wilk test, unpaired *t* test, * *p* < 0.05, ns = not significant. Bar = 10 µm.

Ali et al. have demonstrated the requirement of NAADP-mediated Ca^2+^ signaling in terms of nuclear translocation of the nuclear factor of activated T cells (NFAT) and nuclear factor kappa-light-chain-enhancer of activated B cells (NF-κB) in naïve CD4^+^ T cells [22]. We checked whether Ned-19 could inhibit nuclear translocation of NFAT and NF-κB in anti-CD3-stimulated memory CD4^+^ T cells. Memory CD4^+^ T cells were stimulated with anti-CD3 (10 µg/mL) for 48 h with or without Ned-19, followed by immunohistochemistry analysis. As shown in Figure 7A–C, 50 µM Ned-19 partially inhibited nuclear translocation of NFAT and NF-κB.

## 3. Discussion

NAADP-mediated Ca^2+^ signaling and its effect on downstream effector functions in naïve, effector and natural regulatory T cells has already been reported by Ali et al. [22]. However, this is the first study of NAADP-mediated Ca^2+^ signaling and its impact on the downstream effector functions of primary memory CD4^+^ T cells. We have utilized the NAADP pathway antagonist Ned-19 to investigate various downstream effector functions of Ca^2+^ signaling mediated by NAADP in memory CD4^+^ T cells. 

Ned-19 was discovered via virtual screening as an efficient chemical probe for NAADP. Evidence supports the idea that cell-permeant Ned-19 specifically targets NAADP-mediated Ca^2+^ signaling. In sea urchin egg homogenates, 100 μM Ned-19 was shown to have inhibited NAADP-mediated Ca^2+^ release, but it did not have any effect on Ca^2+^ release caused by either of two messengers, inositol 1,4,5-triphosphate and cyclic ADP-ribose [21]. The competition of Ned-19 with [^32^P] NAADP toward binding to the sea urchin egg binding protein and slow dissociation of bound Ned-19 from the sea urchin NAADP receptor established Ned-19 to be a noncompetitive antagonist of NAADP. The incubation of intact sea urchin eggs with 100 μM Ned-19 in artificial sea water, followed by NAADP injection, did not cause any Ca^2+^ flux. When mouse pancreatic beta cells were incubated with 100 μM Ned-19, followed by application of NAADP (100 nM) using a patch pipette, it inhibited Ca^2+^-dependent current traces, mediated by NAADP [21]. The preincubation of rat uterine smooth muscle cells with 5 μM Ned-19 for 15 min, followed by 400 nM NAADP-AM addition, entirely inhibited the NAADP-mediated intracellular Ca^2+^ surge [30]. Ali et al. have shown that 1 h preincubation of naïve CD4^+^ T cells with 100 μM Ned-19 inhibited the NAADP-mediated Ca^2+^ flux [22]. Much of our approach was pharmacological, using Ned-19 as a probe for NAADP-mediated Ca^2+^ release. 

By using Ned-19, we have demonstrated that for memory CD4^+^ T cells (1) Ned-19 inhibited proliferation in a dose-dependent manner; (2) Ned-19 suppressed cytokine production (partial for IL-2, complete for IFN-γ and IL-10); (3) Ned-19 reduced IL-2 production and thereby intracellular accumulation; (4) Ned-19, between 50 μM and 200 μM, stimulated the TCR/CD3-mediated Ca^2+^ flux and 250 μM–300 μM inhibited the TCR/CD3-mediated Ca^2+^ flux in a concentration-dependent manner; (5) Ned-19 partially reduced NFAT and NF-κB nuclear translocation; and (6) Ned-19 suppressed NAADP-AM-mediated Ca^2+^ flux.

Naïve CD4^+^ T cells proliferate in response to stimulation by anti-CD3 plus anti-CD28, whereas effector and memory CD4^+^ T cells proliferate in response to anti-CD3 alone. Farber et al. have shown that mouse memory CD4^+^ T cells (isolated based on the CD45RB levels, where CD45RB^lo^ cells are memory and CD45RB^hi^ cells are naïve) upon TCR/CD3 crosslinking generated a lesser number of tyrosine-phosphorylated protein species as compared to naïve cells and TCR signaling left ZAP-70 tyrosine kinase in an unphosphorylated and phosphorylated state in memory and naïve CD4^+^ T cells, respectively. So, there are differences in the downstream signaling events between naïve and memory CD4^+^ T cells [31]. Presumably, this is why memory CD4^+^ T cells required 10 μg/mL anti-CD3 stimulation to obtain optimum thymidine incorporation, whereas naïve CD4^+^ T cells required 2 μg/mL anti-CD3 plus 2.5 μg/mL anti-CD28 stimulation in the same assay. Ned-19-mediated inhibition of memory CD4^+^ T cell proliferation raised a question about the possible cytotoxic effect of Ned-19. PMA and ionomycin were able to rescue the proliferation of cells treated with to memory CD4^+^ T cells incubated for one hour with 50 μM Ned-19, but not those treated with 100 μM Ned-19. The synthetic NAADP antagonist, BZ194, was reported to impair proliferation of antigen-experienced CD4^+^ effector T cells. The preincubation times with BZ194 for the optimum inhibitory effect were 8 h and 16 h in Ca^2+^ release and NFAT1 translocation experiments, respectively [23]. With 5 h of preincubation, we did not observe any effect of BZ194 on memory CD4^+^ T cell proliferation, and the same result was reported by Ali et al. with naïve T cells [22]. Our finding is very much consistent with the impaired sensitivity of naïve and long-lived memory T cells to BZ194, as reported by Cordiglieri et al. [32].

It has already been established that TCR activation leads to Ca^2+^ influx, which activates the calcineurin-NFAT pathway, wherein the activated phosphatase calcineurin dephosphorylates NFAT, and as a consequence, NFAT proteins migrate into the nucleus [2]. It has been shown that the IL-2 promoter has NFAT-binding sites and therefore IL-2 expression is regulated by NFAT [33]. Sweetser et al. have demonstrated two strong NFAT-binding sites in the IFN-γ promoter and so NFAT promotes IFN-γ expression [34]. We showed that Ned-19 partially suppressed IL-2 secretion and significantly inhibited IFN-γ and IL-10 secretion by stimulated memory CD4^+^ T cells. The slight reduction (statistically not significant) in IL-2 secretion by Ned-19-treated stimulated memory CD4^+^ T cells as compared to the control was substantiated by intracellular IL-2 staining of these cells in the presence or absence of Ned-19. Ned-19 also partially suppressed intracellular IL-2 production and that too in a dose-dependent manner. 

Sigova et al. have comparatively analyzed naïve and memory T cells (defined as ionomycin-resistant T cells) in terms of the response to Ca^2+^-mobilizing agents, such as Con A, thimerosal, thapsigargin and ionomycin. They observed a dearth of intracellular Ca^2+^ stores in memory T cells and insensitivity of memory T cells to Ca^2+^-mobilizing agents [35]. The above-mentioned findings and differences between naïve and memory CD4^+^ T cells in downstream events of TCR signaling have prompted us to optimize the conventional receptor-mediated Ca^2+^ flux assay for memory CD4^+^ T cells. Memory CD4^+^ T cells required a much higher concentration of biotinylated anti-CD3 (25 μg/mL) as compared to naïve CD4^+^ T cells (5 μg/mL). Thereafter, the study of receptor-mediated Ca^2+^ flux in memory CD4^+^ T cells in the presence of Ned-19 concluded that 50, 100 and 200 μM concentrations of Ned-19 stimulated receptor-mediated Ca^2+^ flux and 250 and 300 μM of Ned-19 exhibited concentration-dependent inhibition of receptor-mediated Ca^2+^ flux. The thapsigargin-mediated Ca^2+^ flux of cells pre-incubated with 300 μM Ned-19 confirmed the viability of the cells. The reduction in the Ca^2+^ flux in the absence of extracellular Ca^2+^ showed that Ca^2+^ influx from the extracellular medium, presumably via store-operated Ca^2+^ channels, contributed to the receptor-mediated Ca^2+^ flux. The complete inhibition of the receptor-mediated Ca^2+^ flux in the case of thapsigargin-preincubated cells substantiated the contribution of the ER Ca^2+^ store to the receptor-mediated Ca^2+^ flux. The partial inhibition of the receptor-mediated Ca^2+^ flux in the case of bafilomycin A1-preincubated cells revealed the partial contribution of lysosome/acidic Ca^2+^ stores in receptor-mediated Ca^2+^ flux. The action of bafilomycin A1 was confirmed by showing the disruption of the pH gradient of the lysosomes or acidic vesicles, as demonstrated by loss of Lysotracker Red staining of bafilomycin A1-preincubated memory CD4^+^ T cells. In addition to this, pre-treatment of memory CD4^+^ T cells with Ned-19 and Lysotracker Red, followed by confocal microscopy, showed weak co-localization in lysosomes or acidic vesicles, indicating the possible location of the Ned-19 target. This also showed that Ned-19 did not disrupt the pH gradient required for Lysotracker Red staining. This result was very much in alignment with a study where NAADP receptors in mouse pancreatic beta cells were fluorescently labeled using Ned-19 [21]. Trufanov et al. have illustrated a great extent of cis-NED 19 and Lysotracker co-localization in rat aorta smooth muscle cells [28].

NAADP, being negatively charged, does not readily enter cells unless it is taken up by a transport mechanism, such as connexin-43 hemichannels [22,36]. Parkesh et al. have discussed NAADP-AM, an acetoxy methyl derivative of NAADP, which could readily go inside the cell and then be converted into NAADP by esterases [29]. In our study, only 2, 1 and 0.5 nM NAADP-AM caused Ca^2+^ flux. Preincubation of memory CD4^+^ T cells with 250 μM Ned-19 suppressed the Ca^2+^ flux caused by all three concentrations of NAADP-AM. However, the activity of different preparations of NAADP-AM was variable, reflecting the instability of the reagent. The viability of cell samples that were incubated for 1 h with Ned-19, followed by the addition of NAADP-AM during the Ca^2+^ flux, was confirmed by the addition of thapsigargin at a later time point, which caused a Ca^2+^ flux. Hence, the presence of Ned-19 did suppress the NAADP-mediated Ca^2+^ flux when NAADP was shown to be present. In reference to the Ned-19-mediated partial inhibition of IL-2 secretion and significant inhibition of IFN-γ and IL-10 secretion by anti-CD3-stimulated memory CD4^+^ T cells, we showed that Ned-19 mediated the partial inhibition of NFAT and NF-κB (not significant) nuclear translocation and therefore could mediate the inhibition of cytokine production by stimulated memory CD4^+^ T cells. If we compare the effect of NAADP-mediated Ca^2+^ signaling in naïve and memory CD4^+^ T cells based on this study and the study conducted by Ali et al. [22], we will conclude that Ned-19 suppresses proliferation, cytokine production, NFAT and NF-κB translocation to the nucleus in both the cells. Ned-19 inhibits Ca^2+^ flux in a dose-dependent manner, with complete inhibition at 100 μM in naïve CD4^+^ cells, whereas in memory CD4^+^ cells, it stimulates Ca^2+^ flux between 50 and 200 μM and inhibits Ca^2+^ flux between 250 and 300 μM. 

In conclusion, NAADP-mediated Ca^2+^ signaling generated via TCR activation has significant effects in terms of proliferation, cytokine (IL-2, IFN-γ and IL-10) secretion, and other downstream effector functions of memory CD4^+^ T cells. The memory CD4^+^ T cells utilized here were generated against antigens present in the environment. In the future, the study of NAADP-mediated Ca^2+^ signaling in memory CD4^+^ T cells generated against a specific antigen would be clinically beneficial and therefore enable us to look for better approaches to manipulate Ca^2+^ flux in memory cells. 

## 4. Materials and Methods

### 4.1. Experimental Animals

Fifteen- to forty-week-old female C57BL/6J mice, obtained from Jackson Laboratory (Bar Harbor, ME, USA), were used for the experiments. The mice were kept under daily supervision in a specific-pathogen-free environment in the animal facility of the University of Toledo Health Science Campus. All the experiments with mice complied with the U.S. NIH guidelines for the Care and Use of Laboratory Animals. The protocol used for the project was approved by the Institutional Animal Care and Use Committee (IACUC). Approval Code: 107556 Approval Dates: 16 October 2014, 14 November 2017, 13 November 2020, 12 November 2023.

### 4.2. Medium

The T cell medium consisted of RPMI 1640 with L-glutamine (Corning Cellgro, Corning, CA, USA), 10% heat-inactivated fetal bovine serum/HI-FBS (Atlanta Biologicals, Minneapolis, MN, USA), 3 × 10^−5^ M 2-mercaptoethanol (Fisher Scientific, Hampton, NH, USA), 2 × 10^−3^ M L-glutamine, pH 7.4, 10 mM HEPES buffer (Sigma Aldrich, St. Louis, MO, USA), 100 U/mL penicillin, 100 µg/mL streptomycin, and 1% media additions (0.06 g folic acid (Gibco), 0.36 g L-asparagine (Gibco, Carlsbad, CA, USA), 1.16 g L-arginine (Hazleton Biologics, Inc., Lenexa, KS, USA), 2.16 g L-glutamine (MP Biomedicals, LLC, Irvine, CA, USA), and 1.10 g sodium pyruvate (MP Biomedicals, LLC) in 100 mL PBS). The memory CD4^+^ T cell medium (used during isolation) consisted of PBS with 2% HI-FBS and 1 mM EDTA. The FACS medium consisted of 0.05% bovine serum albumin (BSA, Fisher Scientific) and 0.1% sodium azide (Fluka, Charlotte, NC, USA) in PBS. The medium for the Ca^2+^ flux experiments using flow cytometry consisted of 2% HI-FBS in Hank’s balanced salt solution (HBSS) with CaCl_2_, MgCl_2_ (Gibco by Life Technologies, Carlsbad, CA, USA).

### 4.3. Memory CD4^+^ T Cell Isolation

The mouse spleen was disrupted using a sterile loose-fitting glass homogenizer in a medium composed of 2% HI-FBS in HBSS without calcium, magnesium or phenol red, followed by filtration through a 70 µm nylon mesh sterile cell strainer (Fisher Scientific). Then, the cells were resuspended in memory CD4^+^ T cell medium after centrifugation, followed by aspirating off the supernatant. The number of nucleated cells in that cell suspension was determined after lysing red blood cells in an aliquot of the cells. This was chosen because RBC Lysing Buffer could not be used with the isolation kit. Finally, after repeated centrifugation, the spleen cell pellet was resuspended in 1 mL memory CD4^+^ T cell medium. Thereafter, 10^8^ nucleated spleen cells were processed using EasySep^TM^ Mouse Memory CD4^+^ T Cell Isolation Kit (Stemcell Technologies, Vancouver, BC, Canada). FACS analysis showed that there were 82.6% CD62L^low^CD44^high^ cells (effector memory CD4^+^ T cells) and 17.0% CD62L^high^CD44^high^ (central memory CD4^+^ T cells). All the memory cell experiments were performed using similarly isolated cells.

### 4.4. Memory CD4^+^ T Cell Proliferation Assays

A flat bottom, tissue culture-treated polystyrene, sterile 96-well plate with a lid (CytoOne, USA Scientific, Ocala, FL, USA) was coated overnight with eBioscience^TM^ anti-mouse CD3 in PBS (10 µg/mL) or PBS. After isolation, memory CD4^+^ T cells in T cell medium (5 × 10^5^ cells/mL) were incubated for 1 h with DMSO or increasing concentrations of Ned-19 in DMSO in the incubator at 37 °C and 5% CO_2_. When using BZ194, the incubation period of the memory CD4^+^ T cells with DMSO or BZ194 was 5 h. The washed plate was seeded with cells (10^5^ cells/well) as per the proliferation plan. In some wells, phorbol 12-myristate 13-acetate (PMA) (100 ng/mL, Sigma-Aldrich) and ionomycin (500 ng/mL, Calbiochem, Burlington, MA, USA) were added to cells already incubated for an hour just before they were seeded into the plate. The plate was then incubated at 37 °C and 5% CO_2_ for 48 h. Thereafter, [^3^H] thymidine (specific activity: 7.0 Ci/mmol and concentration: 1.0 mCi/mL) from Moravek, Inc. (Brea, CA, USA) in T cell medium was added to each well (1 µCi/well) and the plate was incubated at 37 °C and 5% CO_2_ for another 24 h. The cells were harvested onto a filter plate (UniFilter-96 GF/B, PerkinElmer, Waltham, MA, USA). The plate was dried overnight at room temperature. On the next day, the bottom of the plate was sealed and then 40 µL of liquid scintillation cocktail (MP Biomedicals, Irvine, CA, USA) was added to each well. The top of the plate was sealed with a transparent sticker and the plate was read in a Perkin Elmer Top Count NXT.

### 4.5. ELISA Assays (IL-2, IFN-γ, and IL-10)

A flat bottom 96-well plate was coated overnight with anti-mouse CD3 (eBioscience, ThermoFisher, Carlsbad, CA, USA) in PBS (10 µg/mL for IL-2/2 µg/mL for IFN-γ and IL-10 ELISA) or PBS at 4 °C. Isolated memory CD4^+^ T cells in T cell medium (5 × 10^5^ cells/mL) were incubated for 1 h with DMSO, 50 µM, and 100 µM Ned-19 in DMSO at 37 °C and 5% CO_2_. Meanwhile, the plate was washed twice with PBS. Thereafter, 1 µg/mL soluble anti-mouse CD28 (eBioscience) was added to those cell suspensions that would be seeded to wells coated with anti-mouse CD3 (for IL-10 and IFN-γ ELISA). Thereafter, the plate was seeded with cells (10^5^ cells/well) and the plate was incubated at 37 °C and 5% CO_2_ for 48 h (96 h for IL-10 and IFN-γ ELISA). The supernatant was collected and stored in the freezer at −80 °C until the ELISA was performed. The ELISA was performed using IL-2 Mini ABTS ELISA Development Kit/Murine IL-10 Mini TMB ELISA Development Kit/Murine IFN-γ Standard TMB ELISA Development Kit (all from Peprotech, Carlsbad, CA, USA). TMB one-component HRP Microwell Substrate (Surmodics, Eden Prairie, MN, USA) was used as substrate. The plate was read at 620 nm (for IL-2 ELISA)/450 nm with wavelength correction set at 620 nm (for IL-10 and IFN-γ ELISA) using a CLARIOstar microplate reader from BMG Labtech (Ortenberg, Germany).

### 4.6. Flow Cytometry

Immunophenotyping was performed to determine the purity of the isolated cells. A BD Accuri C6 flow cytometer was used for the analysis and the following anti-mouse antibodies from eBioscience were used for immunophenotyping: CD4APC (final concentration 1 µg/mL), CD8a FITC (2.5 µg/mL), CD4 FITC (2.5 µg/mL), CD8a APC (1 µg/mL), CD62L FITC (2.5 µg/mL), and CD44 APC (1 µg/mL). The cell pellet was suspended in Fc block for 10 min, followed by diluted primary antibodies and incubation in ice for 1 h. The cells in FACS medium were washed once through a cushion of HI-FBS and FACS medium was added into the pellets for flow cytometry. All the flow experiments analyzed at least 10,000 cells per group.

For the intracellular IL-2 staining, memory CD4^+^ T cells from 2 spleens (1.75 × 10^6^ cells/mL) were incubated for 1 h with DMSO or increasing concentrations of Ned-19 at 37 °C and 5% CO_2_. The cells were seeded in an anti-mouse CD3 (eBioscience) pre-coated (10 µg/mL) flat-bottom 96-well plate and the plate was incubated at 37 °C and 5% CO_2_ for 6.5 h, followed by 1000× brefeldin A solution (eBioscience) in T cell medium (final concentration in culture: 1:1000) and further incubation of the plate for an additional 7.5 h at 37 °C and 5% CO_2_. The cells were stained first with primary antibody for CD4 glycoprotein, and then fixed and permeabilized using Intracellular Fixation and Permeabilization Buffer Set (eBioscience) following the protocol, followed by staining for intracellular IL-2. The acquisition of the samples was performed in a BD Biosciences FACS Calibur flow cytometer and the data were processed using FCS Express7 Flow Cytometry software. The anti-mouse antibodies from eBioscience used for intracellular IL-2 staining analysis were CD4 APC, CD4 Alexa Fluor 488, IL-2 APC, and rat IgG2b kappa isotype control APC.

### 4.7. Calcium Flux

Calcium flux was determined via flow cytometry. After isolation, memory CD4^+^ T cells (about 10^5^ cells/tube) were taken into 5 mL polystyrene tubes with cap (Evergreen Scientific, Bothell, WA, USA) as per the design of the experiment and washed once with FACS medium for Ca^2+^ flux. Then, the cells were loaded with the acetoxymethyl (AM) ester derivative of Fluo-4 or Fluo-4, AM (1.6 µM) and the acetoxymethyl (AM) ester derivative of Fura Red or Fura Red, AM (3.06 µM) cell permeant fluorescent dyes (Invitrogen by Thermo Fisher Scientific) and incubated at 37 °C and 5% CO_2_ for 30 min. The cells were incubated at 37 °C and 5% CO_2_ with trans-Ned19 (Tocris, BioTechne, Minneapolis, MN, USA) for one hour, thapsigargin (Calbiochem, Burlington, MA, USA) for 30 min, and bafilomycin A1 (LC Laboratories, Woburn, MA, USA) for 30 min. Just before the calcium flux, the cells were washed once with FACS medium for Ca^2+^ flux at room temperature. Calcium flux, defined as the ratio of FL1 to FL3, was conducted using a BD Biosciences FACS Calibur flow cytometer. The population of live cells was gated via forward and side scatter and fluorescence at FL1 (530 nm) and FL3 (670 nm) was monitored. For the TCR-mediated Ca^2+^ flux, the cells were stimulated via addition of 25 µg/mL (final concentration after addition) biotinylated anti-mouse CD3 monoclonal antibody or Anti-Mo CD3e, eBioscienceTM Biotin, functional grade (Invitrogen by Thermo Fisher Scientific) at 50 s followed by 50 µg/mL (final concentration after addition) of streptavidin (Jackson ImmunoResearch Laboratories, Inc., West Grove, PA, USA) addition at 2 min 30 s. For the NAADP-mediated calcium flux of memory CD4^+^ T cells, NAADP-AM (AAT Bioquest, Pleasanton, CA, USA) was added at 1 min. Acquisition was briefly interrupted for each reagent addition. The data were analyzed using FCS Express 7 Flow Cytometry software. 

### 4.8. Imaging of the Lysosomes of Memory CD4^+^ T Cells

Memory CD4^+^ T cells were incubated with 100 µM Ned-19 for 30 min at 37 °C and 5% CO_2_. Then, 60 nM Lysotracker Red DND-99 (Molecular Probes, Fisher Scientific, Hampton, NH, USA), 1 µM bafilomycin A, or DMSO were added and the incubation continued for another 30 min at 37 °C and 5% CO_2_. The cells were plated on glass-bottom microwell dishes (P50G-1.5-30-F, MatTek Corporation, Ashland, MA, USA) pre-coated with poly-L-lysine (100 µg/mL). The images were captured with a Leica TCS SP5 laser scanning confocal microscope equipped with 5 conventional lasers plus multi-photon excitation, which produces laser excitation lines: 458, 488, 514, 561, 633, and a tunable Ti-Sapphire MP laser 710–990 nm. While capturing the Lysotracker Red DND-99-stained images, the 577/590 nm excitation/emission setting was used. The multi-photon laser excitation at 760 nm and the collected emissions at 415–488 nm were utilized during capturing of the Ned-19-stained image. Quantitation was performed using ImageJ 1.54g software.

### 4.9. NFAT and NF-κB Translocation Analysis

Memory CD4^+^ T cells were stimulated by anti-mouse CD3 (10 µg/mL) from eBioscience in the presence of DMSO or 50 µM Ned-19 for 48 h at 37 °C and 5% CO_2_. Then, the cells were transferred onto microscope cover glass (Fisher Scientific), which was coated overnight with poly-L-lysine in water (50 µg/mL) in a flat-bottom with a lid 6-well cell culture plate (Corning Incorporated, Corning, NY, USA). The cell suspension (3.15 × 10^5^ cells/mL for DMSO treated and 2.9 × 10^5^/^mL^ for 50 µM Ned-19 treated sample) was incubated in the wells at 37 °C and 5% CO_2_ overnight. On the next day, the cells were fixed using 2% paraformaldehyde at room temperature for 15 min. After 3 washes with PBS, the cells were permeabilized using 0.3% Triton X 100 for 25 min. After 3 washes with PBS, 1% BSA in PBS was added and incubated for 30 min to 1 h to block any nonspecific binding. Thereafter, mouse monoclonal antibody IgG1 NFATc1 (7A6) sc-7294 and rabbit polyclonal antibody NF-κB p65 (C-20): sc-372 from Santa Cruz Biotechnology (Dallas, TX, USA) were added at 3 µg/mL (final concentration) for immunohistochemical staining of NFAT and NF-κB in permeabilized cells. The cells were kept overnight at 4 °C. On the next day, after washing 3 times with PBS, secondary antibodies Texas Red labeled donkey anti-rabbit IgG (1:1000; Jackson ImmunoResearch) and Alexa Fluor 488 affinipure donkey anti-mouse IgG (1:1000; Jackson ImmunoResearch) were added and incubated for 1 h at room temperature. The cover glasses were mounted onto slides with ProLong Gold Antifade Mountant with DAPI (Molecular Probes). The immunofluorescence images were captured using a Nikon Eclipse Ti microscope with 100× oil objective. The quantification of the images was performed with ImageJ 1.54g software and the quantification of NFAT and NF-κB translocation to the nucleus was performed by taking the ratio of the integrated intensity (the product of the selected area in square pixels and mean gray value) of NFAT or NF-κB to DAPI. The average of 4 cells was determined in the analysis, as very few cells stuck to the cover glasses.

### 4.10. Statistical Analysis

The statistical analysis was performed using GraphPad Prism 10.0.2 software. The normal distribution analysis was conducted with the Kolmogorov–Smirnov or Shapiro–Wilk test. The proliferation and IL-10 and IFNγ ELISAs were analyzed using a one-way analysis of variance (ANOVA) with Tukey’s Multiple Comparison Tests, where **** *p* < 0.0001, *** *p* < 0.001, ** *p* < 0.01, * *p* < 0.1 and ns = not significant. IL-2 production was analyzed using the Kruskal–Wallis test. Lysosome staining and transcription factor translocation were analyzed using an unpaired *t* test, * *p* < 0.05. Ned-19 staining was analyzed using the Mann–Whitney test.

## Data Availability

Data is contained within the article or Supplementary Material.

## Supplementary Materials

The following supporting information can be downloaded at https://www.mdpi.com/article/10.3390/molecules29040907/s1, Figure S1. Flow cytometry gating and staining data for intracellular IL-2 production.
