# Supplementary material for: Nicotinic Acid Adenine Dinucleotide Phosphate (NAADP)-Mediated Calcium Signaling Is Active in Memory CD4+ T Cells
Source: Molecules. 2024 Feb 19;29(4):907. doi: 10.3390/molecules29040907 (PMC10892544; doi:10.3390/molecules29040907)
Supplement: Supplementary file 1 [file molecules-29-00907-s001.zip › molecules-2806332-supplementary.pdf]

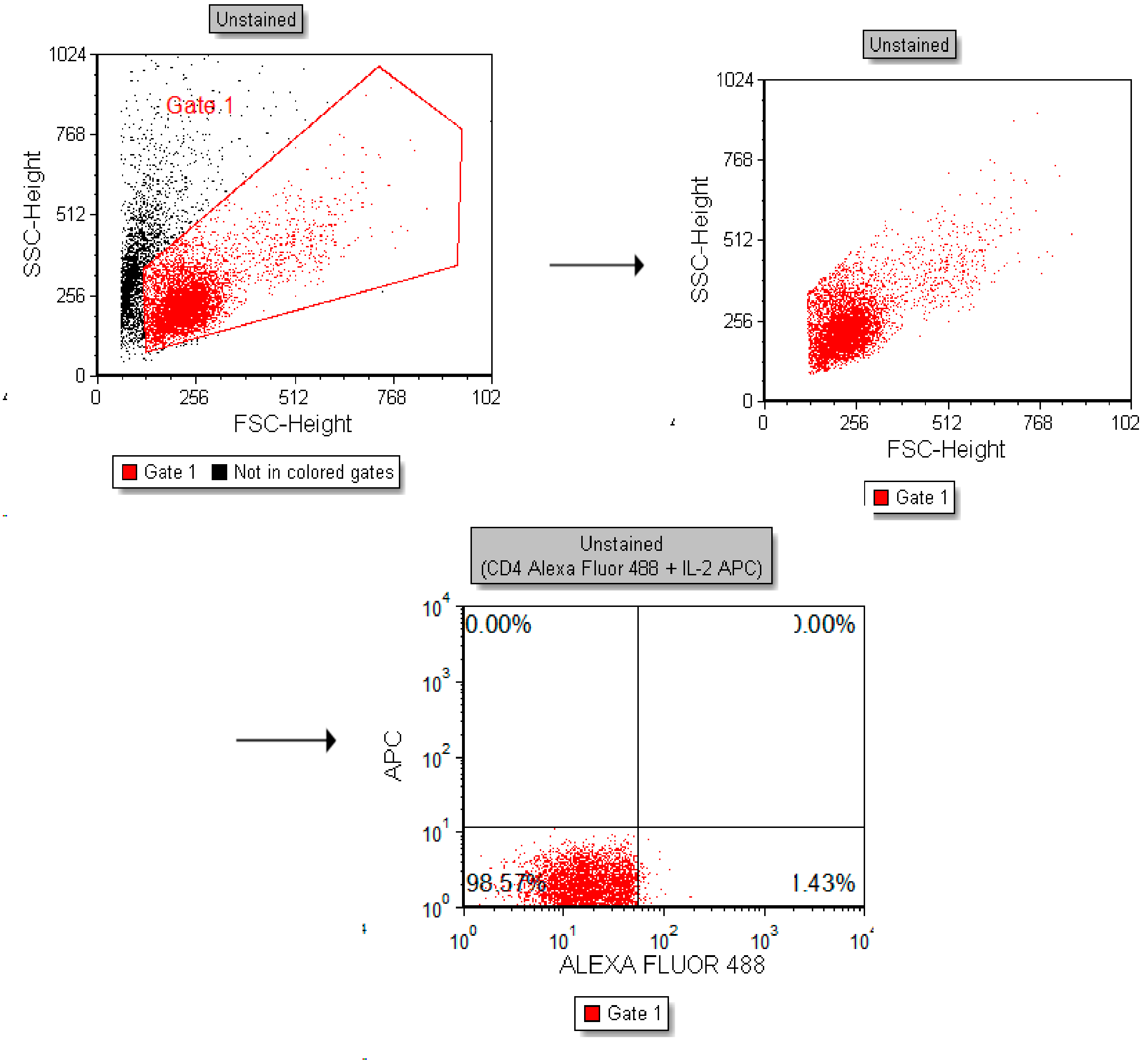
Unstained
1024
768
512
256
0
SSC-Height
Gate 1
0
256
512
768
102
FSC-Height
Gate 1
Not in colored gates
Unstained
1024
768
512
256
0
SSC-Height
0
256
512
768
102
FSC-Height
Gate 1
Unstained
(CD4 Alexa Fluor 488 + IL-2 APC)
APC
0.00%
98.57%
1.43%
ALEXA FLUOR 488
Gate 1

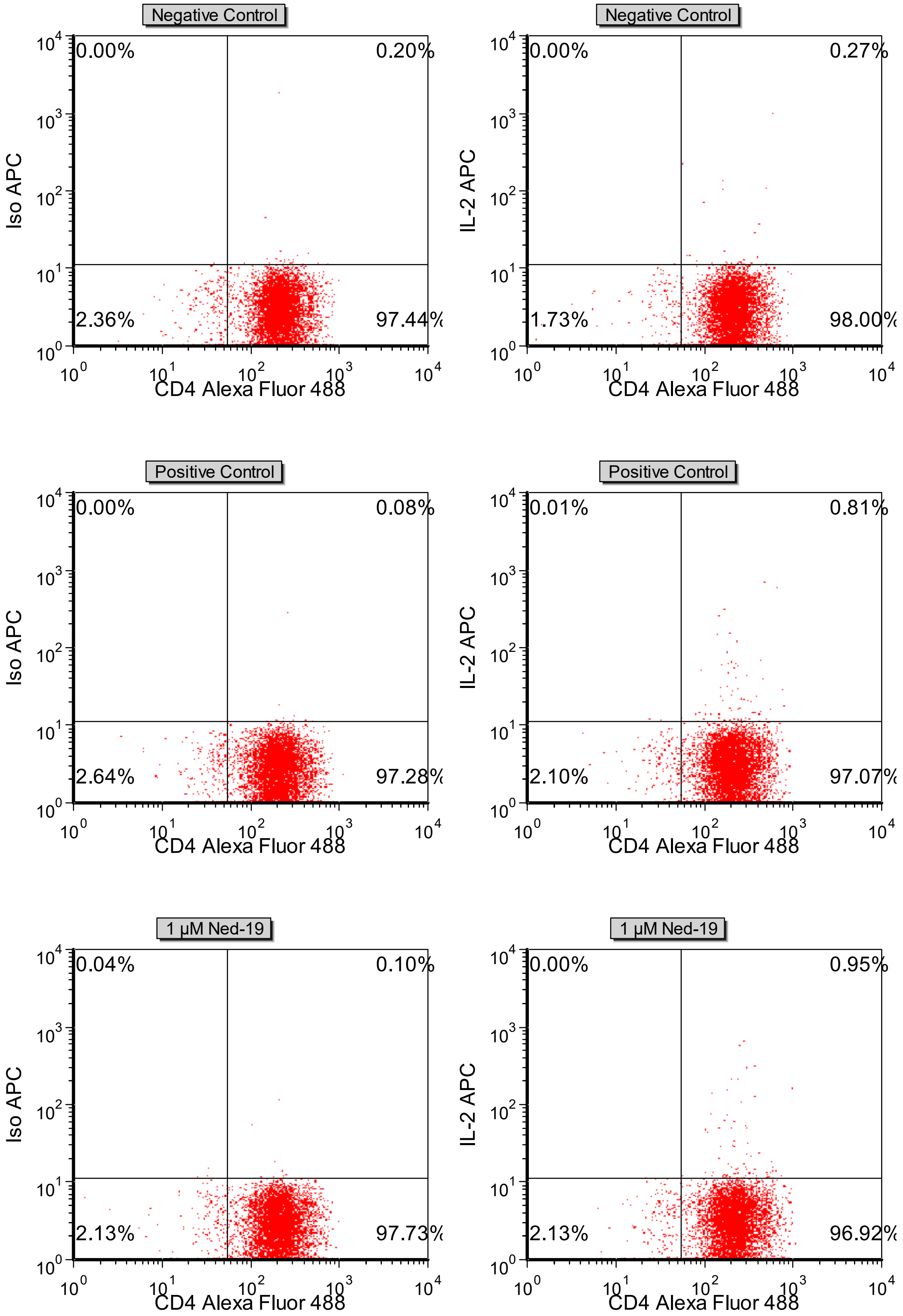
Negative Control
Iso APC
CD4 Alexa Fluor 488
0.00%
0.20%
2.36%
97.44%
Negative Control
IL-2 APC
CD4 Alexa Fluor 488
0.00%
0.27%
1.73%
98.00%
Positive Control
Iso APC
CD4 Alexa Fluor 488
0.00%
0.08%
2.64%
97.28%
Positive Control
IL-2 APC
CD4 Alexa Fluor 488
0.01%
0.81%
2.10%
97.07%
1 µM Ned-19
Iso APC
CD4 Alexa Fluor 488
0.04%
0.10%
2.13%
97.73%
1 µM Ned-19
IL-2 APC
CD4 Alexa Fluor 488
0.00%
0.95%
2.13%
96.92%

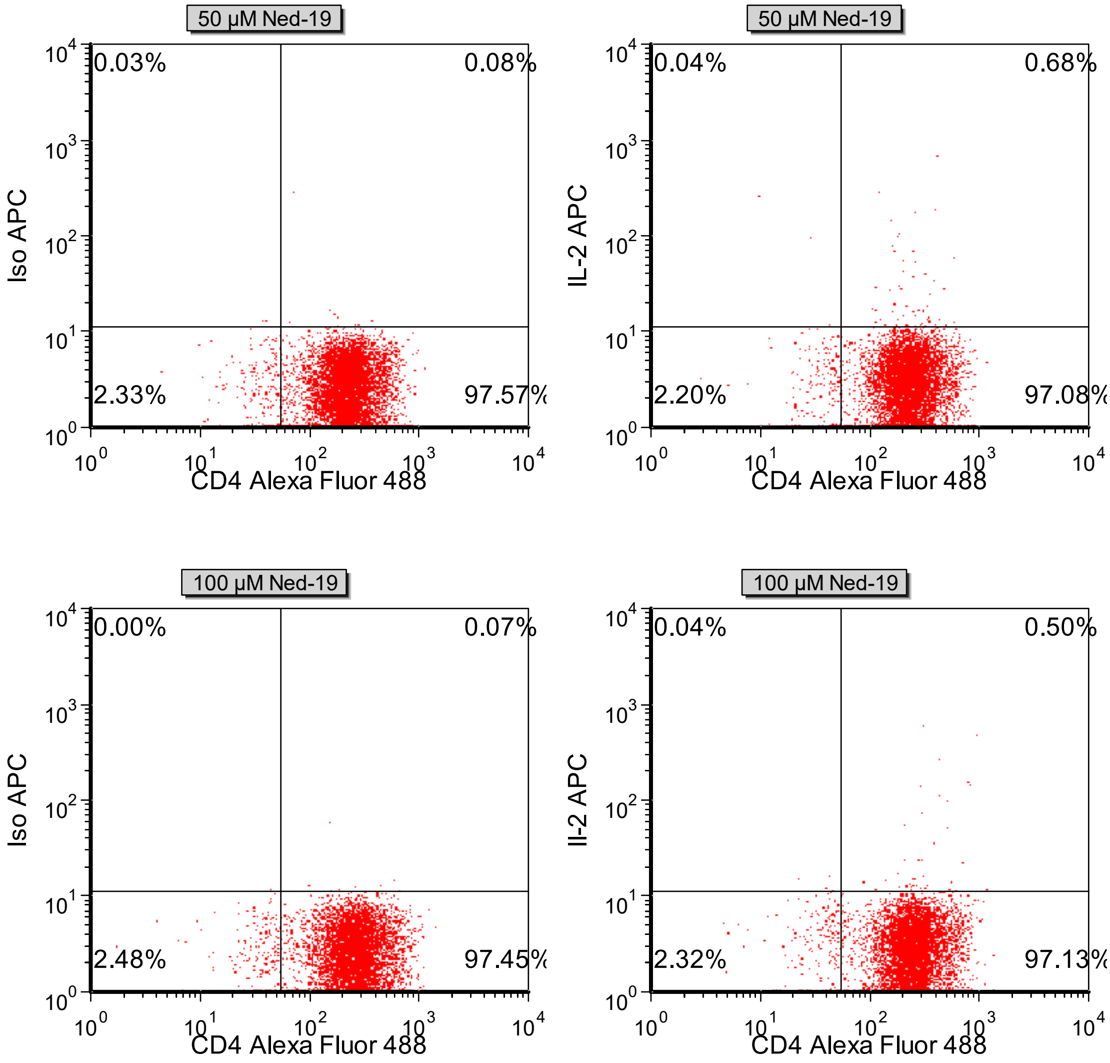

Figure S1. Flow cytometry gating and staining data for intracellular IL-2 production. Memory CD4+ T cells were incubated for 1 hour with Ned-19/DMSO and then cells were stimulated by anti-mouse CD3 for 6.5 hours, followed by addition of 1000x Brefeldin A solution (1:1000) and again cells were stimulated for another 7.5 hours. Thereafter intracellular IL-2 staining was done after fixation and permeabilization with anti-CD4 AlexaFluor 488 and APC anti-mouse IL-2 or APC isotype control. Negative control cells were not stimulated by anti-CD3.
